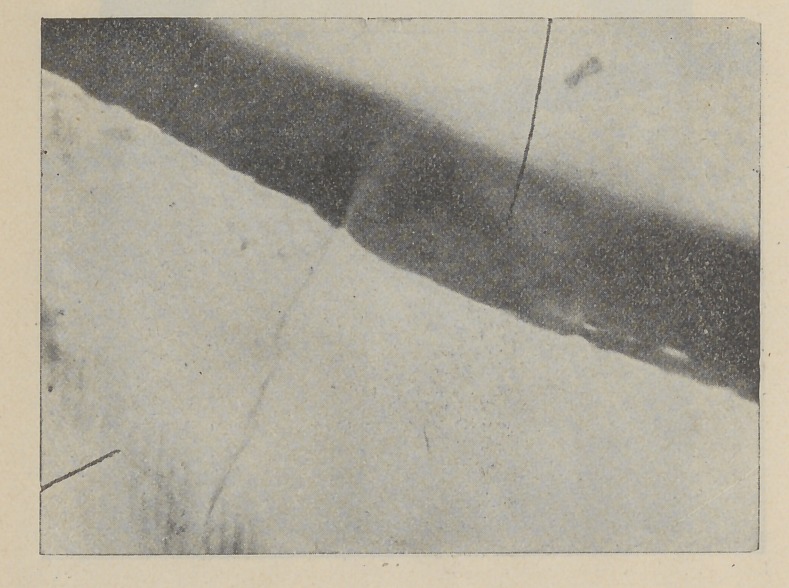# Evolution of Decay

**Published:** 1899-12

**Authors:** Arch C. Hart


### Selections.

### Evolution of Decay.

ARCH C. HART, D.D.S.

I believe decay to be a natural force which acts through media upon all material bodies, with the effect of changing their identity. I do not consider it a process of a force, nor as the result of the action of a form of energy, but as a force or form of energy that is only known to us by its manifestations through matter—effecting changes from a state of soundness or perfection to one less sound or perfect. I would classify it on the same general basis as gravitation, or any of the other great forces of nature. Gravitation existed and man used its power long before Newton formulated the law of gravitation. Men did not understand how the force acted.

So with the force decay. For ages men have recognized and used this power and depended upon its action for their existence. In these living bodies are we not dying to live, and living to die? But to prove that this force acts with other forces in making men and worlds grow old would be difficult. The lack of demonstration, however, makes it no less a fact. All things are growing or decaying; advancing in integration or disintegration. The change from perfection to one less perfect, to my mind, results from the action of a force, and we term it decay. Surely life is a force. Why then is not decay a force?

Fire, air, light electricity, acids, alkalies, salts, alcohols, oils and water I take to be some of the important media through which decay acts in effecting change of identity seen in material bodies. In citing water as one of the most important media I have done so because it is one of the most universal of all compounds as well as the greatest of nature's solvents and cements. Science has already proven that upon water for many of their combinations depend the animal, the vegetable and even the mineral kingdoms. The cell holds imprisoned within its walls water of combination.

The crystal holds within its angles water of crystalization. I do not think any argument is necessary to prove the importance of water as a medium for the action of decay.

In explanation of the action of many chemical compounds, chemistry, to my mind, teaches that many of the acids, alkalies, salts, alchohols and oils are antiseptics or germicides chiefly owing to their relative affinity for water. To say a substance is germicidal because it kills germs is like calling pistols homicidal because they kill men. Such an answer is not sufficiently scientific. How do antiseptics and germicides act as a class? What are the principles involved? Bacteria, we have already learned, will not grow on tissues that have been treated with solutions of certain strength of bichloride of mercury, nitrate of silver, chloride of gold, sulphate of copper, chloride of tin, formaldehyde, the essential oils, and many of the alcohols, acids and alkalies. For example, in the treatment of gonorrheal ophthalmia, the cause of as much blindness as any one disease, after having thoroughly cleansed the eye by repeated douchings of lukewarm sterilized water, there is instilled several drops of a two percent solution of nitrate of silver, which, if used in time, will suffice to stop the disease.

How these chemicals act so as to prevent the growth of bacteria is due to their ability to harden albumen and render it insoluble to the action of bacteria; they were powerful in preventing decay just in proportion to their ability to form insoluble albuminates with the various tissues of the body.

How they harden albumen and render it insoluble is explained as follows: Chemistry teaches us that nitrate of silver and the list of germicides already named, likewise alcohol, acids, alkalies and oils, are constantly demanding water. That in many instances when applied to tissues they cause a shrinkage or expansion beyond recognition. That the changed appearance noted in the tissue is due, in part at least, to the altered condition of the water in the tissues. The nitrate of silver, for example, has so changed the water of combination that the bacteria can not penetrate the film thus formed with the tissues, and the bacteria in the tissue have either had sufficient water removed from them to cause their death, or else have become so confined that they can not get the water necessary for their proper growth. The soil has in reality become hardened, insomuch as it is now insoluble to their digestive action, and might with truth be called antiseptic.

In proof of the importance of rendering the water in the tissues inaccessible to bacteria, and that the way the cells render themselves germicidal is by removing the water from the bacteria themselves, or else so placing the water of the tissues as to be insoluble to the digestive action of bacteria, it is necessary to explain why foods are preserved from the action of bacteria by cooking, drying, freezing, salting, sugaring, alcohol, acids, alkalies and oils.

In cooking foods, boiling, frying, baking, etc., the water is driven out of them, so that they shrink quite beyond recognition. Bacteria, when present, of course, are generally killed, and those that may get lodgment do not grow well, owing to the decreased quantity of water now present in the tissues. Freezing solidifies the water in the tissues, and while they remain frozen the water is inaccessible to bacterial growth. Drying removes the water of combination in part, so although the tissues may be covered with bacteria, the bacteria do not grow; not because the bacteria are dead, but because they can not get the water necessary for their growth. The proof that it is only water that is needed, any one knows who has had experience with dried foods that have become damp. In the use of smoke as applied in curing hams, bacon, etc., the heated air removes water from the tissues, and in its penetration carries into the tissues creasote and the other active agents that have an affinity for water. The creasote, etc., removes the water from the tissues, and especially from the surface, and because of a more immediate contact forms a layer insoluble to bacteria. Salting or sugaring are similar. The salt abstracts water from the tissues. Salt is sprinkled over fish as they are packed in vessels for preservation without the addition of any brine, as they are said to make their own brine—the salt literally squeezes the water out of the bodies of the fish. Any child knows that eating sugar will make him thirsty.

When we eat salty foods or much sweets we remove the water from our tissues, in reality cause a fever and thirst, just as if we were ill with some disease characterized by an increase of temperature. The sugar or salt enters the tissues and removes the water in part, and this increases the demand on the part of the tissues for more water. So with alcohol.

Bacteria, we have already learned, are only able to thrive when liberally supplied with water. There are other conditions necessary, as temperature, rest, light, etc. I mention water, as it is one of the conditions I believe we are able to control sufficiently to prevent their growth.

When a patient is down with typhoid fever or any of the fevers that owe their development to bacteria, the patient suffers with thirst and fever. Why? I believe it is because the growth and development of bacteria is consuming such enormous quantities of water that the system is over-drained, and the patient, if his tissues have not sufficient vitality to hold the water in combination, dies often simply because the bacteria have used up all the available water.

Physiologists speak of cells becoming specialized in their work of elaboration in the body. They mean that the cells divide up the work of the body just the same as a body of men divides up the work of a community. By reason of this division of labor they are able to do their work better; they are specialists. To illustrate: The liver is an organ whose cells are specialized in the secretion of bile, glycogen, etc.; while the periosteum might be called an organ having cells specialized in the secretion of bone-forming materials. So when the bones of the old are fractured they do not readily knit, because the cells specialized in the secretion of bone-forming compounds have almost entirely disappeared. It is the ability on the part of cells to become specialists, capable of doing one thing extremely well, that imparts to the organism immunity from certain diseases. The cells, learning that bacteria tend to remove the water from them, endeavor to fix the water in themselves. They may do this by the elaboration of some chemical compound that has a greater affinity for the water of combination than have the bacteria. Be that as it may, the bacteria can not get the water necessary for their growth.

I believe if it is through a change in the main compound of the body that it becomes possible for disease to manifest itself, whether it be an atrophy or hypertrophy of the tissues resultant from the action of bacteria, chemical or natural forces, the more stable we can fix, seal or combine the water in the tissue, the more effective will be its power of resisting decay.

From my experiments with the teeth I believe it is possible to fix, seal or combine the water in the teeth so as to be inaccessible for a time to bacteria.

It has already been proved that certain conditions did confer immunity for a time to the digestive action of bacteria; that some teeth were less susceptible than others; that decay ofttimes is checked and the layer of decalcified dentin rendered immune to the further action of bacteria; that while the cracks in the enamel and the spaces between the cells are large enough for the entrance of bacteria there is often no decay of sufficient importance to attract attention, unless the enamel or dentin be examined by the aid of the microscope and differential stains. Then the surface enamel and dentin will show bacteria adhering, and even penetrating for a considerable distance the structure of the tooth.

The above figure shows the penetration of nitrate of silver into enamel partly decomposed by bacteria. *a*, Immune layer of enamel deeply stained. Taken from a young lady's mouth in which white decay was rapidly destroying the teeth. This tooth was treated three years ago to nitrate of silver without filling. Decay stopped in all teeth thus treated. Tooth crowned.

The extent of decomposition thus produced by bacteria may be as I have stated only visible through the microscope or differential staining. (See fig. 1, a : and fig. 2).

For example, when a tooth presenting the white chalky spots of beginning decay is treated with a solution of nitrate of silver, the extent of decay can be readily seen.

This partially decomposed surface containing millions of bacteria, I believe, can be made to take up substances capable of forming a layer immune to bacterial growth. So I have given the term "immune layer" to any layer of enamel or dentin that has become sterile to the growth of bacteria. This layer is partly due, in some mouths, to a protective film of bacteria that has become stained, and remains adherent to the surface of the teeth.

Section of enamel showing immune layer that has been produced by the action of formaldehyde or formalin upon the enamel. Hardened in alcohol 50 percent, formalin 10 percent while still warm. Stained with Rubin Seosin and oil of cinnamon. The staining brings out the depth of penetration or the fact of bacteria. Under direct sunlight the bacteria can be seen penetrating the enamel at points indicated.

Figure 3 is the same section as figure 2, under a higher power. Shows coating of acid-producing and enamel-destroying bacteria, on top of figure; on lower side depth of penetration, stained by eosin.

I have observed many mouths in which there was no decay, or where decay had ceased; and occasionally getting such teeth for minute examination, I have found the outside layer of enamel and dentin to be stained with various compounds. The exact chemical nature of these compounds I have not been able to prove to my entire satisfaction. On scraping off this protective stain, the microscope reveals it to be chiefly bacteria, and because of this I am inclined to believe that certain bacteria have the power of protecting the teeth, just as lichen growing on rocks protects them from disintegration.

This shows penetration of tobacco stain in the enamel. Tooth lateral incisor from man aged fifty-three. Has used tobacco since he was a boy. Mesio-approximal cavity with tooth-structure black from infiltration of stain. Cavity has been there for years, never became any larger, so he never had it filled. Tooth evidently immune to the action of bacteria.

The bacteria causing this peculiar discoloration I have observed growing near the gum margin, and in places difficult of cleansing, especially on the lingual and approximal surfaces. Their growth on the teeth will be noticed as a peculiar black or reddish line, and when present it is quite impossible to find decay. From this it would seem that there are certain bacteria which of themselves have the power to confer immunity from attacks of those bacteria that cause the destruction of the teeth.

In mouths where the decay is slow the decayed portion is always discolored. The cause of this discoloration is an important question, and in many mouths one quite difficult of solution. In many instances I have found the discoloration is tobacco stain. This, I observed, had penetrated quite deeply the enamel and dentin, and, by the aid of direct sunlight reflected by the mirror up through the Abbe condenser, and the specimen under the microscope, one could see many bacteria which had taken on the stain from tobacco. This would suggest that the discoloration seen in decay is partly due to bacteria having become stained.

The use of direct sunlight in the examination of specimens stained to show bacteria will make visible bacteria as far as they have penetrated the tooth structure. This idea came from observing how a ray of sunlight would make visible the bacteria and particles of dust in a dark room. I immediately made use of it in my study of specimens of teeth under the microscope. It was not necessary to grind them so thin to show bacteria penetrating both enamel and dentin.

This immune layer is sometimes due to the cracks and spaces in and between the cells having become filled with insoluble compounds, as is seen in teeth stained with tobacco or nitrate of silver, chloride of gold, sulphate of copper, etc., similar to the manner that oil covers the surface of water and retards its evaporation. In other words, the water already in the enamel, dentin and bacteria has attracted compounds that have a greater affinity for the water in the tooth substance than have the bacteria. So decay stops for a time, simply because the bacteria can not grow down in the enamel, not because the hole is too small, but because the water in the tooth has become too insoluble or inaccessible to the digestive action of bacteria. The remains of the dead bacteria actually serve as a filling and protection against the entrance of other bacteria.

We carry into actual practice the application of the principle, viz: protecting the water in enamel and dentin by causing the tooth, and the bacteria as far as they have penetrated the tooth, to take up certain substances dissolved in water, like formaldehyde, nitrate of silver, chloride of gold, sulphate of copper, chloride of zinc, chloride of tin, and a whole host of substances of a like nature that I believe have the power of protecting the water in the tissues from the growth of bacteria.

Formaldehyde is used about as follows: After cleansing the surface to be hardened with pyrozone (three percent medicinal) make several applications of the formaldehyde, varying in strength from two to forty percent (forty percent being full strength as it comes to us from the shops) to the cavity, carious surface, and healthy portion of the tooth and teeth under the rubber dam, from ten to forty minutes. The cavity is then dried out and coated with a saturated solution of paraform in chloroform, to which has been added sufficient hard Canada balsam to make the solution a thin varnish. Into this, after waiting for the varnish to dry, may be burnished amalgam, stuck gold, gutta percha or cement.

Formaldehyde should never be applied to the surfaces of the teeth, except the rubber dam be in position, fitted evenly around the necks of the teeth, so that there shall be no holes whereby the mucous surfaces of the mouth may become exposed to the action of formaldehyde, as it produces an ugly slough. In the application of the formaldehyde it is important to have the surfaces of the teeth free from all adhering colonies of bacteria, so as to be sure to kill all bacteria that have penetrated the enamel or dentin.

When once the people fully realize that bacteria alone are the cause of their soft, chalky teeth, and that it is possible to so harden the teeth that they will resist the solvent action of bacteria, then the gilded and glaring signs, "painless extraction," will have to come down. Dentistry will become a profession loved by mankind, and to be called a dentist, an honor.—*Pacific Med. Journal.*

## Figures and Tables

**Figure f1:**
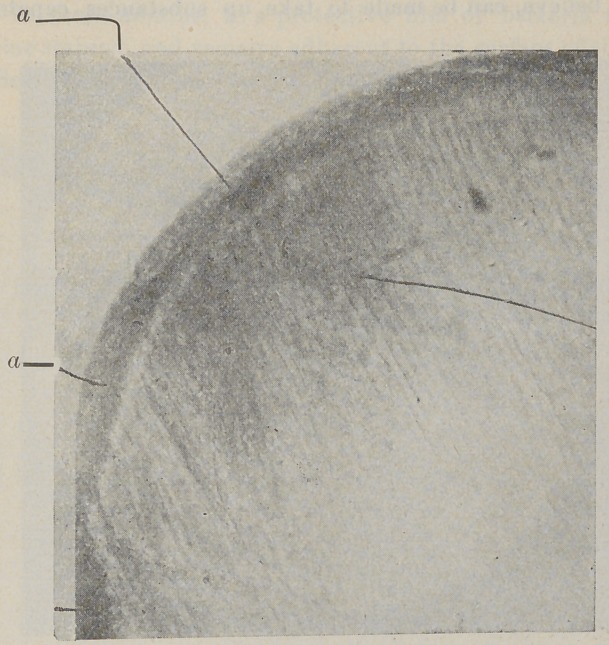


**Figure f2:**
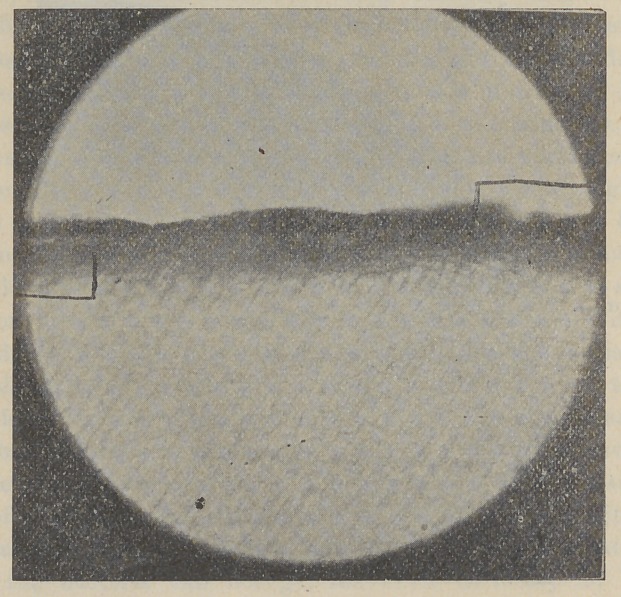


**Figure f3:**
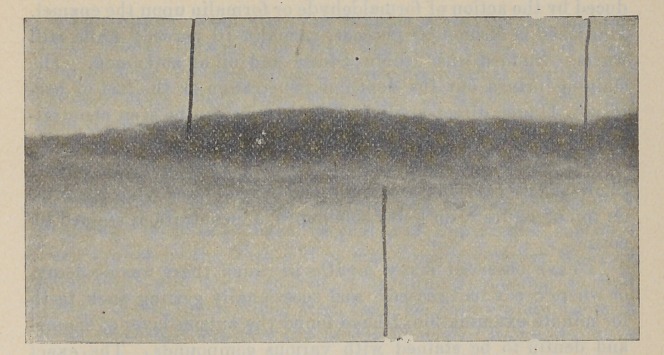


**Figure f4:**
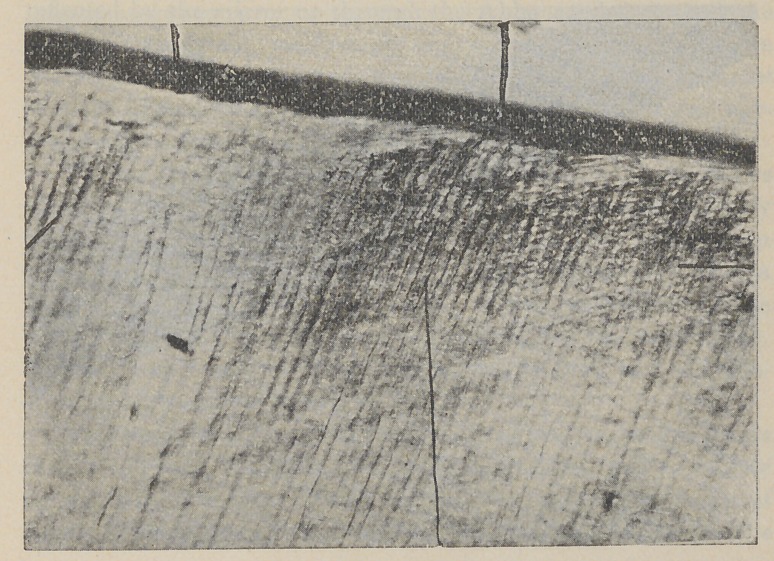


**Figure f5:**